# Prediction of the Sleep Apnea Severity Using 2D-Convolutional Neural Networks and Respiratory Effort Signals

**DOI:** 10.3390/diagnostics13203187

**Published:** 2023-10-12

**Authors:** Verónica Barroso-García, Marta Fernández-Poyatos, Benjamín Sahelices, Daniel Álvarez, David Gozal, Roberto Hornero, Gonzalo C. Gutiérrez-Tobal

**Affiliations:** 1 Biomedical Engineering Group, University of Valladolid, 47011 Valladolid, Spain; martaferpoy@gmail.com (M.F.-P.); daniel.alvarez.gonzalez@uva.es (D.Á.); robhor@tel.uva.es (R.H.); gonzalocesar.gutierrez@uva.es (G.C.G.-T.); 2 Centro de Investigación Biomédica en Red en Bioingeniería, Biomateriales y Nanomedicina (CIBER-BBN), 47011 Valladolid, Spain; 3 Electronic Devices and Materials Characterization Group, Department of Computer Science, University of Valladolid, 47011 Valladolid, Spain; benja@infor.uva.es; 4 Office of The Dean, Joan C. Edwards School of Medicine, Marshall University, 1600 Medical Center Drive, Huntington, WV 25701, USA; gozal@marshall.edu

**Keywords:** central sleep apnea, obstructive sleep apnea, abdominal respiratory signal, thoracic respiratory signal, convolutional neural network, deep learning

## Abstract

The high prevalence of sleep apnea and the limitations of polysomnography have prompted the investigation of strategies aimed at automated diagnosis using a restricted number of physiological measures. This study aimed to demonstrate that thoracic (THO) and abdominal (ABD) movement signals are useful for accurately estimating the severity of sleep apnea, even if central respiratory events are present. Thus, we developed 2D-convolutional neural networks (CNNs) jointly using THO and ABD to automatically estimate sleep apnea severity and evaluate the central event contribution. Our proposal achieved an intraclass correlation coefficient (ICC) = 0.75 and a root mean square error (RMSE) = 10.33 events/h when estimating the apnea-hypopnea index, and ICC = 0.83 and RMSE = 0.95 events/h when estimating the central apnea index. The CNN obtained accuracies of 94.98%, 79.82%, and 81.60% for 5, 15, and 30 events/h when evaluating the complete apnea hypopnea index. The model improved when the nature of the events was central: 98.72% and 99.74% accuracy for 5 and 15 events/h. Hence, the information extracted from these signals using CNNs could be a powerful tool to diagnose sleep apnea, especially in subjects with a high density of central apnea events.

## 1. Introduction

Sleep apnea is a very common respiratory disorder that affects a large proportion of the adult population worldwide. It is estimated that 9–38% of adults suffer from some degree of sleep apnea [1], with the exact percentage varying depending on factors such as sex, age, obesity, or other co-existing disorders [2,3]. People with sleep apnea experience either pauses or reductions in airflow while sleeping [4]. These respiratory events can last from 10 s to several minutes, with these events recurring throughout the night. From an etiological point of view, there are three main types of apneic events: obstructive, central, and mixed [4]. Obstructive events are the most common and occur in the presence of increased upper airway collapsibility, leading to upper airway resistance or complete obstruction in the presence of ongoing respiratory effort. In central events, however, there is no respiratory effort as brain centers underlying respiratory commands stop their activity due to a variety of mismatched chemosensory and motor drives. Finally, mixed events are a combination of both obstructive and central events. These definitions highlight the importance of the respiratory motion information to determine the type of apneic events [4].

Sleep apnea leads to disrupted sleep patterns, intrathoracic pressure fluctuations, hypoxemia, and hypercapnia [5], ultimately predisposing the affected subjects to substantial morbidities, such as cardiometabolic, cancer, and neurodegenerative dysfunctions [6,7,8]. However, despite these increased risks, sleep apnea remains a relatively underdiagnosed disease [9,10]. One reason is the relatively unavailability of the gold standard diagnostic test, the overnight polysomnography (PSG) [11]. A PSG is a conglomerate of measurements during the night that assess brain activity, breathing, heart rate, and cardiac electrical activity, and other vital signs during sleep. While the reliability and dependability of the PSG are widely recognized, it entails spending a night in a hospital or outpatient clinic and involves the recording of a large number of biomedical signals, rendering it an intricate and expensive test that demands specialized staff [12,13]. The overnight recordings are then analyzed off-line to determine the presence and severity of sleep apnea by estimating among several other metrics the apnea–hypopnea index (AHI), which accounts for the rate of apneic and hypopneic events per hour of sleep [4]. Therefore, PSG is all together labor-intensive, expensive, and time-consuming. This fact, together with the high prevalence of the disease, causes sleep units to become frequently overwhelmed, thereby leading to long waiting lists and delays in both diagnosis and treatment [14].

To deal with this unfavorable situation, the scientific community is seeking automated diagnostic alternatives that simplify sleep apnea detection. These efforts are usually based on the analysis of a reduced set of physiological signals. In this regard, cardiorespiratory signals, such as airflow (AF), blood oxygen saturation (SpO_2_), and electrocardiogram (ECG), have been comprehensively evaluated in the literature [15,16,17]. In this study, we propose using the information contained in the thoracic (THO) and abdominal (ABD) movement signals for this very same purpose. These PSG-derived signals have not been comprehensively appraised, perhaps due to the intrinsic instability of the sensors used for their acquisition [18,19]. However, the American Academy of Sleep Medicine (AASM) establishes that THO and ABD signals are essential for distinguishing between central and obstructive apneic events [4]. It has been observed that thoracic and abdominal movements occur simultaneously when breathing is normal [18,20]. Conversely, respiratory movement amplitude increases during obstructive events, except that thoracic movements occur in the opposite direction to abdominal movements, in an attempt to overcome the obstruction, a phenomenon also termed “paradoxical breathing” [18,20]. As mentioned above, these movements are reduced or even nullified in the case of central respiratory events [18,20].

Based on these considerations, Ng et al. (2008) investigated the utility of mean absolute amplitude from THO and ABD to distinguish obstructive or central events from normal breathing [21]. Lin et al. (2017) evaluated the amplitude ratio, frequency ratio, and correlation between both signals to differentiate among normal breathing, obstructive events (including mixed events), and central events [18]. Other studies conducted this classification relying on the time-domain phase difference [20], instantaneous phase, and phase-locking value of THO and ABD [22]. The information derived from the wavelet analysis of these signals was also evaluated [23,24,25], even combined with wavelet features extracted from AF and/or SpO_2_ to automatically score apneic events or classify them according to their origin [26,27,28,29]. All these studies focused on characterizing the THO and ABD behaviors by means of a conventional feature-engineering approach, thus requiring the extraction of suitable features and relying on the potentially limited knowledge on the effects of sleep apnea in overnight THO and ABD signals. Accordingly, the limited abstraction level of these methods may have hindered their ability to successfully discern complex patterns [30].

Deep learning techniques emerge as a suitable alternative as they allow for the automatic use of relevant features and patterns regardless of whether these features are already known [16,30]. Particularly useful are convolutional neural networks (CNNs) for this purpose. In addition to providing computational efficiency and transfer learning capabilities, CNNs can identify local features, learn high-level hierarchical representations, and recognize them even if they appear in a different location [30,31]. To the best of our knowledge, deep learning methods, such as the long short-term memory (LSTM), have already been used with respiratory movement signals [32,33], but only the previous studies of Wang et al. (2023) and Haidar et al. (2018) applied CNN to these parameters in this context [33,34]. However, their models were more complex since the investigators also used information from the AF signal. Furthermore, all of the aforementioned studies focused on an event classification problem, thus suffering from drawbacks associated with the election of the window size where these events are being sought. In contrast, our study proposes the use of a two-dimensional (2D) CNN architecture capable of predicting the AHI for each subject following a regression problem from the number of apneic events contained in 20-min signal segments. Thus, we aimed to demonstrate that THO and ABD are sufficient to estimate the AHI and preserve their accuracy in predicting central respiratory events.

Thereby, the main contribution of our research was the development and validation of a novel CNN-based deep learning model to automatically extract all relevant information from THO and ABD signals related to apnea events. This model was tested on a large sample of 5000 THO and ABD recordings, showing high diagnostic capacity compared to conventional feature engineering methodologies that used information from these signals to detect apnea events. Additionally, our proposal improved the results from the only found state-of-the-art study that applied techniques of deep learning to them for estimating the AHI. Moreover, and to our knowledge, this is the first time that intermediate AHI thresholds (1, 5, 10, 15, and 30 events/hour (e/h)) have been established to comprehensively evaluate central sleep apnea by jointly using THO and ABD with CNNs.

In the following sections, we detail the materials and methods used for our research (Section 2), which encompass the database used (Section 2.1), the data preprocessing and augmentation conducted (Section 2.2), the architecture and training of our CNN (Section 2.3 and Section 2.4, respectively), the AHI regression (Section 2.5), and the metrics applied for the statistical analysis (Section 2.6). Afterwards, we present our research findings in Section 3, where the results obtained in the optimization of the CNN model (Section 3.1) and in the evaluation of its diagnostic performance (Section 3.2) are collected. Subsequently, Section 4 is dedicated to a comprehensive discussion of our findings and their implications, as well as some recommendations for future research. Finally, we conclude our study in Section 5, summarizing the key conclusions and contributions.

## 2. Materials and Methods

### 2.1. Database

We used the polysomnographic data obtained from the Sleep Heart Health Study database (SHHS, ClinicalTrials.gov Identifier: NCT00005275), which is accessible through the National Sleep Research Resource (https://sleepdata.org/datasets/shhs, accessed on 20 July 2023) [35,36]. In this study, a total of 6441 subjects (5722 valid studies) participated in the initial stage (SHHS1) from November 1995 to January 1998. In a subsequent stage (SHHS2), 2651 of these subjects (2535 valid studies) underwent a second PSG between January 2001 and June 2003. The study was approved by the Ethics Committee of the participating centers, and the subjects involved gave their informed consent.

All these subjects were diagnosed following the rules of the AASM [4]. The common AHI thresholds 5, 15, and 30 e/h were used to determine sleep apnea severity (no apnea: AHI < 5 e/h; mild: 5 e/h ≤ AHI < 15 e/h; moderate: 15 e/h ≤ AHI < 30 e/h; severe: AHI ≥ 30 e/h). Table 1 shows a summary of their sociodemographic data. As Karhu et al. (2021) previously pointed out [37], there are inconsistencies between the annotations of respiratory events (central apneas, obstructive apneas and hypopneas, desaturations, arousals, etc.) and the clinical indices provided with the sleep studies. Consequently, we have taken as a direct reference the events annotated by the clinicians rather than the indices provided as variables.

**Figure 1:**
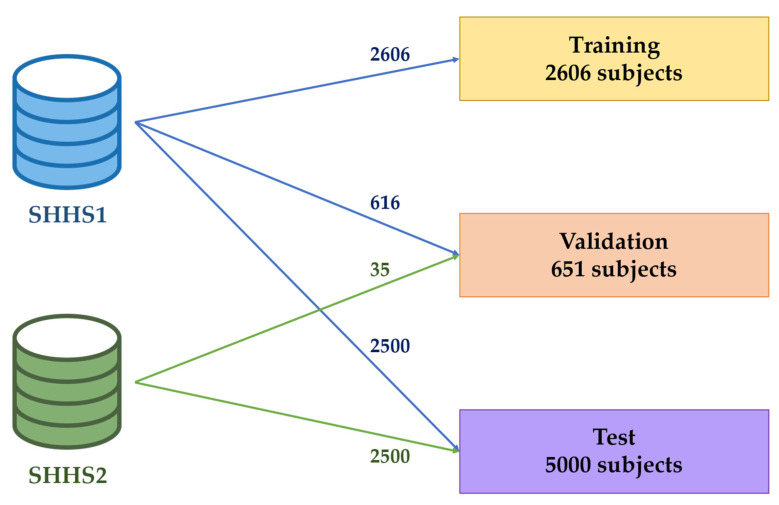
Data distribution in training, validation, and test sets.

As shown in Figure 1, the subjects were allocated into three groups: training, validation, and test sets. As previously mentioned, the patients involved in SHH2 had previously been part of SHH1 in an earlier stage. Using signals from these patients in different groups could potentially introduce bias into the results. In order to mitigate this fact, all signals from those patients who had participated in both SHHS1 and SHH2 were include only in the test set. The remaining instances were randomly allocated to the training set (80% of this remaining) and the validation set (20% of this remaining) [38].

All PSGs were conducted at the patients’ homes with a Compumedics P-Series Sleep Monitoring System. During this test, THO and ABD signals were recorded using inductive plethysmography bands at a sampling rate of 8 Hz (35 signals from SHHS1) and 10 Hz (8222 signals from SHHS1 and SHHS2). As different sampling frequencies were present, we decided to include only those respiratory movement signals obtained at 10 Hz. Consequently, 35 instances from SHHS1 were excluded from our study and their corresponding 35 follow-up signals from SHHS2 were assigned to the minority set, i.e., the validation set.

The regression analysis was based on the number of apneic events (obstructive and/or central events) contained in each signal segment. When an apneic event spanned across 2 different segments, the time covered in each of them was proportionally assigned based on its duration. This allowed for a more accurate representation of event distribution in the segments.

### 2.2. Preprocessing and Data Augmentation

THO and ABD signals were filtered to remove potential artifacts that could negatively affect the quality of predictions performed in subsequent stages of our study. To this effect, a Finite Impulse Response (FIR) low-pass filter with a cutoff frequency of 1.5 Hz and a Hamming window was applied, thus minimizing possible phase distortions or ripples within the passband. The signals were segmented into 20 min intervals, as this duration has proven useful for detecting clustered apnea and hypopnea events [39,40]. Each segment was z-score-standardized to normalize the baseline levels of THO and ABD. After heuristic trials, data augmentation was applied to increase the number of samples in training and validation sets by generating 20 min signal segments with a 75% overlap [41].

### 2.3. Convolutional Neural Network Architecture

**Figure 2:**
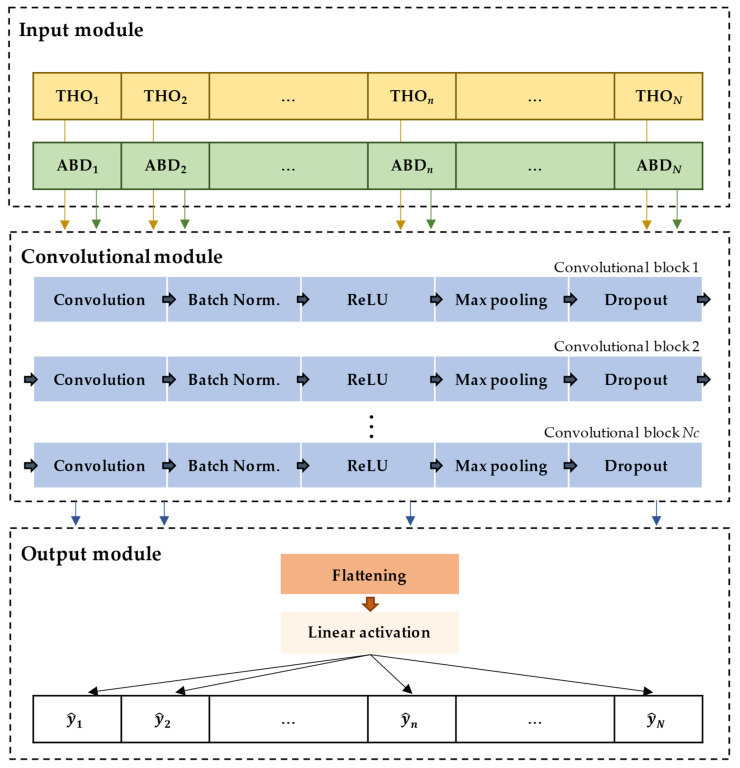
Architecture of the convolutional neural network (CNN) used in this study.

The proposed model is a custom CNN architecture, which was designed to tailor to the specific problem we address in our study. While the layer type and order of this architecture were inherited from previous works carried out in our research group to estimate the severity of pediatric OSA using SpO_2_ and/or AF signals [39,42], the remaining CNN components were customized. As shown in Figure 2, our approach consists of three modules. The first module is the input module, which receives the standardized 20 min segments of THO and their corresponding 20 min segments of ABD. In this module, segments are handled as two stacked dimensions, i.e., the two signals are treated as a matrix of 2 × 12,000 samples, where 12,000 corresponds to the length of the 20 min segments (calculated as 20 min × 60 s/min × 10 Hz) instead of two separate signals. The data then proceeds to the convolutional module of *Nc* convolutional blocks, where *Nc* is a parameter to be optimized. Each of these blocks is composed of the following consecutive layers [30,31]:

- Convolution: It applies a kernel function to the input data to obtain feature maps [30,31]. This layer is defined by three parameters: kernel, padding, and stride. In our study, a padding value of 0 was assigned to prevent artificial expansion of the input data borders, thus maintaining consistent input and output lengths [30,43]. Regarding the stride, it was set to (1, 2) to ensure that the convolutional process preserves the spatial relationships within the data while efficiently extracting meaningful features, leading to a more accurate representation of the input data in the subsequent layers of the network [30,43]. In addition, the number *Nk* and size *Sk* of the kernel were values to be optimized.
- Normalization: This layer normalizes the feature maps using a batch normalization [30,44]. As the network learns, the input distribution of each layer may change, hindering training. Batch normalization mitigates this effect by normalizing the inputs for each channel, subtracting the batch mean, and dividing it by the batch standard deviation. Thus, this normalization not only stabilizes and accelerates the training process but also allows for more efficient learning across all layers of the network [43].
- Non-linear activation: It allows the network to learn complex and non-linear relationships from the feature maps [30,31]. We used the Rectified Linear Unit (ReLU) function for this purpose, which assigns zero to all negative values and keeps positive values unchanged [39,42].
- Pooling: This layer reduces the feature maps size by extracting dominant and translationally invariant features [30]. Particularly, we applied a max pooling of (1, 2) with stride (1, 1) [39,42], which divides the feature map into overlapping regions of (1, 2) and selects the maximum value from each region.

Dropout: It is used as a regularization technique to prevent overfitting by reducing interdependence between neurons [30,31]. To do this, a subset of neurons is randomly deactivated during training. The proportion of neurons deactivated in each iteration ($p_{drop}$) was a parameter optimized in our study.

After the *Nc* convolutional blocks, the data feeds the output module that integrates the following layers:

- Flattening: It concatenates all feature maps elements into a single dimension array to allow the next layer to process the data [30].
- Linear activation: This fully connected layer has a single neuron that receives the activations from the previous layer and performs a linear combination of them to conduct the regression task [30]. The output of this perceptron is the estimation of the number of events contained in each 20 min segment [45].

### 2.4. Convolutional Neural Network Training and Optimization

The CNN training process begins with the initialization of the network weights and biases. We used the He-normal initializer, which adjusts the initial values based on the number of neurons in the preceding layers using a normal distribution with specific standard deviation [46]. These weights and biases were then updated by means of the Adam algorithm [47]. Based on the first- and second-order moments of the gradient of the loss function, this algorithm enables faster convergence towards the global minimum by adapting the learning rate to automatically adjust to different gradient scales. In this study, the Adam algorithm used an initial learning rate of 0.001, a batch size of 32, and a total of 200 epochs. As for the error function, we applied the Huber loss defined by the following equation [48]:

(1) $$L_{\delta }=\left\{\begin{array}{c} \frac{1}{2}\cdot {\left(y-f\left(x\right)\right)}^{2}\;\;\;\;\;for\;\left|y-f\left(x\right)\right|\leq \delta \\ \delta \cdot \left(\left|y-f\left(x\right)\right|-\frac{1}{2}\cdot \delta \right),\;\;\;\;\;otherwise \end{array}\right.,$$

where *L_δ_* represents the Huber loss, the parameter *δ* determines the point at which the loss transitions from quadratic to linear behavior, *y* is the actual number of apneic events, and *f*(*x*) is the predicted value by our model. This function combines the characteristics of the mean squared error and the mean absolute error, providing a less sensitive response to outliers in the data [48]. Its *δ* parameter was set to 1, since it is an appropriate choice for random distributions [48].

The architecture CNN proposed in this study was implemented in Python (version 3.8.10) with the open-source machine learning library PyTorch (version 1.13.0 + cu116). All experiments were monitored through CometML (version 3.31.16).

### 2.5. Apnea-Hypopnea Index Regression

In our study, two different CNN models were designed. One model was trained to estimate the global AHI encompassing all types of respiratory events: obstructive and central apneas and hypopneas. Thus, the output label of the CNN was the total number of events contained in each 20 min segment. The second model was developed to determine the central AHI. Therefore, the CNN output label was the number of central respiratory events within the 20 min segment of THO and ABD. From the value reported by the CNN for each segment, the global and central AHI per subject were obtained as the sum of events per segments contained in the recording divided by the sum of the duration in hours of the segments contained in the recording. Subsequently, a linear regression model was adjusted following the next equation:

(2) $${AHI}_{i}=\left(\beta \cdot {AHI}_{{CNN}_{i}}\right)+\varepsilon ,\;\;\;\;\;\;\;i=global,\;central,$$

where ${AHI}_{{CNN}_{i}}$ is the global or central AHI estimated for each 20 min segment, β is the slope coefficient, and ε is the intercept of the linear regression model obtained by the ordinary least squares (OLS) method. Both β and ε were fitted using the training set. The purpose of this linear regression is to correct the CNN’s tendency to under or overestimate the AHI. One of the key reasons for a possible underestimation is that the total recording time used to compute the AHI is greater than the PSG-derived total sleep time, thus reducing the events/hour ratio [49]. Moreover, CNN might tend to overestimate hypopneas by detecting respiratory effort reductions that are not accompanied by oxygen desaturations ≥ 3% or arousals. After this regression process, all estimated values of global and central AHI were categorized following the severity groups described in Section 2.1.

### 2.6. Statistical Analysis

To evaluate the diagnostic performance of the CNN, several metrics were computed. The intraclass correlation coefficient (ICC: the proportion of total variability attributed to differences between predicted and actual data compared to variability within the dataset itself), root mean square error (RMSE: the average magnitude of the differences between predicted and actual data values), Bland–Altman plots (graphical representations of the differences between paired measurements against their mean) [50], and scatter plots (graphical representations of predicted values versus actual values) were used to assess the agreement between the AHI values predicted by the neural network and the actual AHI values obtained by medical specialists using the PSG data. Cohen’s kappa (*kappa*: the level of agreement between observers or evaluators, considering the agreement that might occur by chance) [51], a confusion matrix with four classes (a comprehensive view of the true positives, true negatives, false positives, and false negatives obtained for each class), and overall accuracy (Acc_4_: the proportion of overall subjects rightly classified considering all classes) were obtained as measures to evaluate the agreement between the actual sleep apnea severity classes and those classes derived from our AHI estimations. Furthermore, binary approaches were also assessed according to the common sleep apnea severity thresholds using the following statistical metrics: sensitivity (Se: the proportion of rightly classified subjects with sleep apnea), specificity (Sp: the proportion of rightly classified subjects without the disease), accuracy (Acc: the proportion of overall subjects rightly classified), positive (PPV: the proportion of positive test result which are true positives) and negative (NPV: the proportion of negative test result which are true negatives) predictive values, as well as positive (LR+: the proportion of subjects with SAHS rightly classified with respect to the proportion of healthy subjects wrongly classified) and negative (LR-: the proportion of subjects with SAHS wrongly classified with respect to the healthy subjects rightly classified) likelihood ratios.

## 3. Results

### 3.1. Convolutional Neural Network Optimization

The CNN architectures, as illustrated in Figure 2, were optimized using training and validation sets. The first one allows the CNN to learn and extract meaningful features from the data, while the second architecture was used separately to evaluate the performance of the models when using different hyperparameters. The optimized hyperparameters were $N_{c}$, $S_{k}$, $N_{k}$, and $p_{drop}$. The complete search space, i.e., the range of possible values that were considered and explored in this study to optimize the hyperparameters of our CNN, is presented in Table 2. Regarding the hyperparameter *N_c_*, increasing the number of convolutional layers improves the CNN performance since lower layers detect simple features, while deeper layers learn to recognize more complex patterns [30,43]. Thus, adding more layers typically increases the capacity of the model to capture intrinsic particularities of the data, which is advantageous when dealing with complex and high-dimensional datasets [30,43]. However, it is important to note that there is an optimal *N_c_* value from which deeper networks require more data and computational resources. Furthermore, the input dimension rapidly decreases as the number of layers increases, so the data size may become too small to be useful in additional layers. Consequently, we conducted trials with the values 6 and 8 for this hyperparameter, which are typically used in this context [39,42]. Common *S_k_* values (16, 32, 64, and 128) were also evaluated to achieve the right balance between capturing fine-grained details (typically obtained with smaller *S_k_*) and broader patterns (commonly obtained with larger *S_k_*) [30,43]. This experimentation was particularly significant when considering smaller *S_k_* values for the deeper layers of the CNN due to the progressively reduced dimensions of the input data as it propagates through the layers of the CNN. Furthermore, we varied $N_{k}$ values from 16 to 128. Having a higher $N_{k}$ could empower the network to learn a more extensive array of features, potentially encompassing the diverse intricacies present in the dataset [30,43]. In contrast, an excessive $N_{k}$ might lead to computational inefficiency and overfitting, where the model learns noise instead of meaningful patterns. Thus, its optimization helped in fine-tuning the architecture of our model to not only preserve essential details but also ensure efficient computation. Finally, typical $p_{drop}$ values (from 0.1 to 0.4 in steps of 0.1) were assessed to find a balance between preventing the network from becoming overly reliant on any specific neurons or connections (thus promoting independence) and maintaining the capacity to capture meaningful patterns within the data [30,43]. Consequently, this exploratory analysis enhanced the ability of our model to discern complex patterns, contributing significantly to its overall performance and effectiveness in capturing the underlying structure of the data.

**Figure 3:**
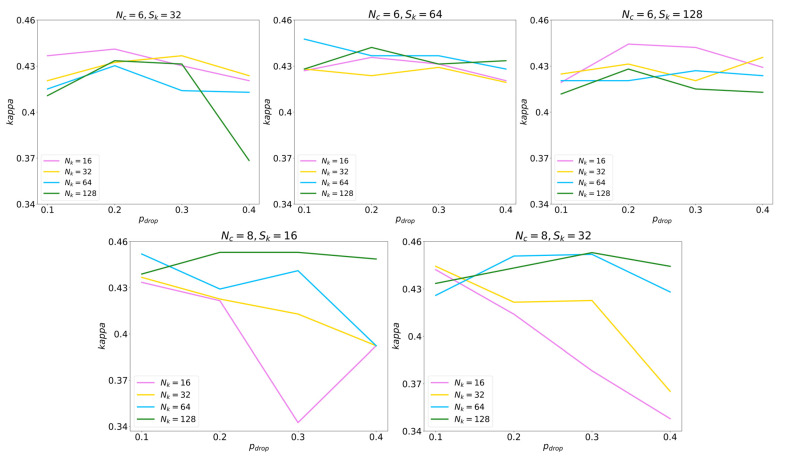
*Kappa* values obtained in the validation set during the CNN hyperparameter optimization process.

In order to assess the performance of each validation experiment, the Cohen’s kappa index was used. This metric provides an evaluation of the trained models, aiming to identify the model with the highest concordance (maximum *kappa*) between the estimated and the actual values of the sleep apnea severity groups in the validation set. Table 2 also shows the selected values for each hyperparameter, based on the best *kappa* achieved. As can be seen in Figure 3, the optimal kappa value obtained in the validation set was 0.453 with $N_{c}$ = 8, $S_{k}$ = 16, $N_{k}$ = 128, and $p_{drop}$ = 0.3.

During the training process of each experiment, the model with the highest kappa in the validation set over the course of the 200 training epochs was chosen as the optimal model. The primary objective was to ensure continual improvement and stability in the models’ performance while also mitigating overfitting.

### 3.2. Convolutional Neural Network Diagnostic Performance

**Figure 4:**
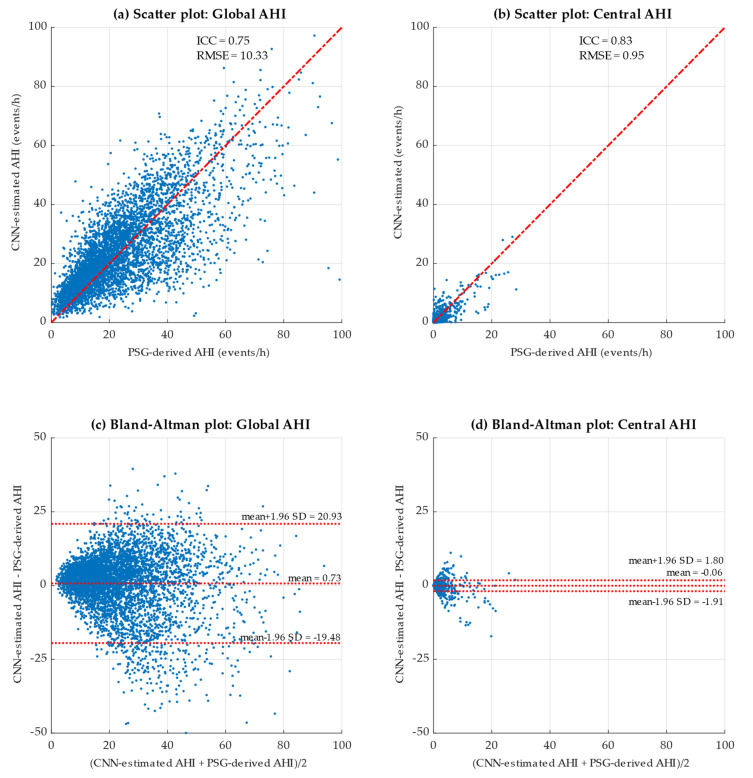
Scatter and Bland–Altman plots obtained by comparing the PSG-derived AHI and the CNN-estimated AHI in the test set considering (**a**,**c**) all apneic events (Global AHI) and (**b**,**d**) the central apneic events (Central AHI).

A combination of scatter and Bland–Altman plots were used in the present study to assess the agreement between the global and central AHI derived from PSG and those estimated by our CNNs in the test set. The results are presented in Figure 4. In the scatterplots of both the global AHI (Figure 4a) and the central AHI (Figure 4b), it could be observed that there was a high proportion of points clustered around the diagonal line, indicating a high agreement between the actual values and those estimated by the CNN. This fact was also reflected in the Bland–Altman plots, where mean values are close to 0. These findings suggest a low bias in both cases, although slightly lower when estimating the central AHI (Figure 4d) than when estimating the global AHI (Figure 4c). Moreover, our proposal achieved an ICC = 0.75 and a RMSE = 10.33 e/h estimating the global AHI in the test set, and ICC = 0.83 and RMSE = 0.95 e/h estimating the central AHI.

**Figure 5:**
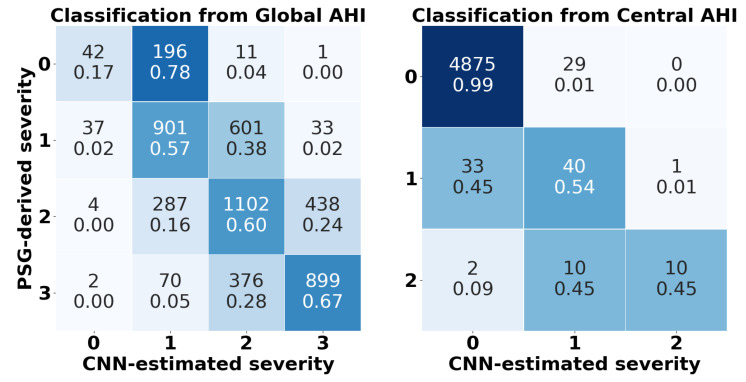
Confusion matrices obtained by the CNN models for the severity prediction from the global and central AHI in the test set, being 0: no apnea; 1: mild apnea; 2: moderate apnea; 3: severe apnea (no subjects with ≥30 central e/h).

The confusion matrices obtained by the predictive model in the test set are presented in Figure 5, along with Table 3 that summarizes the statistical metrics derived from them. This provides an overview of the diagnostic performance of the CNN proposed for the severity prediction from the global and central AHI. The complete AHI model obtained a *kappa* = 0.3960 and accuracies of 94.98% for 5 e/h, 79.82% for 15 e/h, and 81.60% for 30 e/h in the test set. These results improved for the central model as follows: *kappa* = 0.5903, 98.72% Acc for 5 e/h, and 99.74% Acc for 15 e/h (note that there are no subjects with ≥30 central e/h). Although these thresholds are commonly used to evaluate the complete AHI, no similar thresholds are defined in the literature for central events. In order to evaluate our central model according to a realistic number of the latter, we used 1 e/h, 5 e/h, 10 e/h, and 15 e/h as thresholds, reaching accuracies of 94.10%, 98.72%, 99.64%, and 99.74%, respectively. In addition, the confusion matrices and the diagnostic performance metrics separately obtained for SHHS1 and SHHS2 with our model have been included as Supplementary Materials to examine intrasubject variability. Due to the treatment, subjects exhibited a lower severity of global AHI in SHHS2. In contrast, there was an increase in the number of subjects with central apnea that could be attributed to the coexistence of other cardiac pathologies [52]. Thus, our model obtained better overall performance with SHHS1 when estimating the global AHI (*kappa* = 0.3962 and Acc_4_ = 59.56%) and with SHHS2 when estimating the central AHI (*kappa* = 0.6333 and Acc_4_ = 98.40%).

## 4. Discussion

We have developed two 2D-CNN models that were trained, validated, and tested using more than 8000 THO and ABD overnight signals in the context of sleep apnea diagnosis. Both showed high agreement when estimating global and central AHI and exhibited high diagnostic performance when deriving a sleep apnea severity from these estimations, with the central apnea model achieving substantially better performances. This was observed not only when evaluating common AHI thresholds but also when we established intermediate intervals to particularly assess the presence and severity of central apnea (1, 5, 10, and 15 e/h).

Throughout the study, multiple experiments were conducted to optimize the 2D-CNN hyperparameters. Several important findings emerged from these experiments. First, it was observed that increasing the number of layers in the CNN architecture enhanced model performance. A higher number of convolutional layers may increase the model´s capacity to learn complex patterns and representations from the data. Thus, each layer could learn and extract features in breathing patterns associated with apnea and hypopnea events. It is also important to note that smaller kernel size in each convolutional layer of the models enables the capture of local details and fine features in the behavioral changes occurring in THO and ABD. By focusing on smaller regions, the model can detect specific patterns and structures more accurately. Furthermore, the use of a higher dropout has been found to be effective in reducing overfitting. By introducing randomness and disabling a percentage of neurons during training, dropout reduces the dependence between neurons. This regularization technique improves the model´s ability to generalize to unseen examples, as well as enhances learning robust features. Thus, these findings support the hyperparameter configuration we obtained for estimating the severity of apnea based on respiratory effort signals.

As previously mentioned, the scatter plots showcased in Figure 4 reveal a remarkable level of agreement between the actual global AHI and central AHI values and their corresponding estimations. This agreement is evidenced in the tendency observed in these graphs, where the data points predominantly align along the diagonal line. This fact suggests a positive relationship between the estimated and actual values for both cases. The low dispersion presented in the points on the graph further implies that this relationship is highly consistent. Thereby, the limited dispersion within the scatter plot points indicates the robustness of the predictive capability of our model for both global AHI and central AHI. Furthermore, Bland–Altman plots also provide a visual assessment of the agreement between the estimated and actual global and central AHI values. These plots revealed a low bias in both models, as evidenced by the mean difference (central horizontal red line of the Bland–Altman plot). Particularly, this mean indicates the bias in the differences between the values estimated by our model and those derived from PSG. Its value was close to zero for both global and central AHI, suggesting an absence of systematic bias between the methods, i.e., the estimated and actual values exhibit agreement. The Bland–Altman plots also depict the limits of agreement by the upper and lower horizontal red lines. These lines specify an acceptable range of differences between estimated and actual values. As observed, most of the points fall within these limits for the global AHI (Figure 4c: 297/5000 out-of-bounds observations) and central AHI (Figure 4d: 172/5000 observations outside the limits). Moreover, it is noteworthy that the bias is slightly lower when estimating central events, supported by the ICC values obtained for the global AHI (ICC = 0.75) and central AHI (ICC = 0.83) estimations. These ICC values suggest a strong agreement between the predictions and their actual values for both the global and central AHI. The ICC for central events demonstrates an approximately 10% higher agreement compared to predictions for all types of apneic events. Similarly, RMSE values of 10.33 e/h and 0.95 e/h for global AHI and central AHI estimation show a substantial difference in the magnitude of errors between these two predictions. This situation could be conditioned by a greater presence of obstructive events. It is also important to note that RMSE is sensitive to outliers, and the considerably higher RMSE for global AHI estimation compared to central AHI estimation may be influenced by the presence of a small group of outliers.

Looking at the confusion matrix of the global AHI estimation in Figure 5, the model exhibits a higher tendency to misclassify healthy patients, often labelling them as having a mild degree of apnea. Furthermore, a smaller proportion of patients with severe apnea are occasionally classified into less severe apnea groups. Examining the central AHI confusion matrix, the model accurately classifies healthy patients, with a notable number of true positives. However, there are some misclassifications present in mild and moderate groups, where instances are occasionally assigned to adjacent severity levels. Both the global AHI and central AHI confusion matrices reveal no glaring cases where patients with severe apnea are predicted as healthy or *vice versa*, despite some uncertainty in consecutive severity groups. When assessing the performance of the model in the test set using overall metrics, the global AHI estimation achieved a *kappa* of 0.3960 and a multi-class accuracy of 58.88%, indicating a moderately high level of agreement. In contrast, the central AHI estimation reported remarkably higher performance, with a *kappa* of 0.5903 and a multi-class accuracy of 98.50%, revealing a high level of agreement. Although, the Acc achieved for the global AHI at 5, 15, and 30 e/h were higher, a large imbalance was observed in the Se-Sp pair for 5 e/h (99.09% Se–16.80% Sp). A low Sp value obtained for this threshold would indicate that our model overestimates severity in subjects without apnea. Concretely, 17% of the subjects who do not suffer from the disease would be correctly classified, while 78% would be assigned to mild apnea degree. Similarly, a slight overestimation occurred in cases of mild apnea (38% classified as moderate) and moderate apnea (24% classified as severe). It should be noted that these deviations from our model prediction are directed towards adjacent severity class. In contrast, the CNN would be underestimating the severity of central apnea (45% of subjects with 5 e/h ≤ central AHI < 15 e/h classified as without apnea, and 45% of subjects with 15 e/h ≤ central AHI < 30 e/h classified as mild apnea). These results could be conditioned by two factors. On the one hand, there could be a bias towards the majority class, i.e., 5 e/h ≤ central AHI. Although data augmentation was applied, it might not have been sufficient to correct the large imbalance in the dataset used to evaluate the central AHI. On the other hand, 5, 15, and 30 e/h are the thresholds commonly used to assess global AHI, but similar thresholds are not defined in the literature for central events. In this regard, we have not only considered these AHI thresholds, but we have established intermediate intervals to provide a comprehensive evaluation of central apnea severity. Thus, our method obtained a 94.10% Acc when evaluating the presence of central apnea for 1 e/h. Moreover, the CNN achieved remarkably high diagnostic accuracies using the thresholds 5, 10, and 15 e/h (98.72%, 99.64%, and 99.74%, respectively). This also obtained a high Sp for all thresholds (from 97.70% to 99.98%), as well as moderate Se (from 45.45% to 68.57%). As a result, the underestimation was reduced by segregating the thresholds. In addition, the high multi-class accuracy and the high LR+ values (greater than 10) obtained at these thresholds provided strong evidence to establish the presence of each degree of severity [53]. 

Accordingly, these results highlight the strong ability of the model to accurately predict the central AHI values, while demonstrating relatively moderate performance in estimating the global AHI. This variable diagnostic performance can be attributed to the distinctive physical characteristics of human breathing and apneic events. In the case of central apnea, the cessation of breathing occurs without any respiratory effort from the patient. This results in an abrupt disruption in THO and ABD signals. Additionally, the primary cause of breathing interruption in obstructive apnea is the obstruction of the upper airways. It can result in ongoing respiratory efforts and movements of the chest and abdomen, making obstructive apnea events more challenging to distinguish from normal breathing based on these signals. During obstructive events, there is often a paradoxical respiratory movement, characterized by an increased thoracic movement in the opposite direction to abdominal movements, in an attempt to overcome the obstruction [18,20]. However, not all obstructive events exhibit this paradoxical movement; some patients may even manifest normal inspiratory effort during obstructed breaths [18,54]. Consequently, the distinct physiological features and breathing patterns associated with central apnea events are comparatively easier for the CNN to detect when analyzing respiratory signals. Moreover, although respiratory efforts have certain particularities during obstructive events, this information may not be sufficient when the obstructions reflect increased resistance rather than complete obstruction and require additional criteria, such as the presence of oxygen desaturations and/or arousals, to be scored. Hence, these particularities could be conditioning the CNN performance for identifying and classifying the different types of apneic events.

Past studies also focused their research on the individual or joint analysis of these respiratory signals. As can be seen in Table 4, most of these studies aimed at detecting apneic events using methods based on common feature-engineering, reaching accuracies ranging between 84.42% and 95.87% [18,20,21,22,29]. Other studies applied these techniques to differentiate between obstructive, central, and mixed events, obtaining accuracies in the range of 75.85–95.47% [23,24,25,26,28]. It should be noted that the low abstraction level of the methods used in these studies might hinder their ability to successfully discern complex patterns, and even introduce redundant and irrelevant information into their models. This fact, along with the small sample size used in all of these studies (from 6 to 100 subjects), considerably limits the generalizability and, therefore, the ability of their proposal to become relevant and widely applicable. However, we used a large database (8257 sleep studies), which reduced bias. Moreover, we applied advanced deep learning techniques to identify local features of THO and ABD, learn high-level hierarchical representations, and recognize apnea-caused changes, even if they appear at a different location within the segment. In this regard, the preliminary study conducted by Haidar et al. (2018) used CNNs with respiratory movement signals [34]. Their event classification-based model also used information from AF signal, achieving an accuracy of 83.50%. Moreover, deep learning techniques were also applied to THO, ABD, and AF signals in the recent study of Wang et al. (2023) [33]. They reported accuracies between 81.56% and 83.90% using CNN, LSTM, 1D-CNN-LSTM, and 2D-CNN-LSTM. Nevertheless, all these studies were based on the detection/differentiation of apneic events, such that more direct comparisons with our subject-oriented model are difficult.

Additionally, our results also outperformed those obtained by Van Steenkiste et al. (2019) to estimate the AHI by means of an LSTM neural network fed with ABD signals from SHHS [32]. In this case, where we can actually conduct a direct comparison, our proposal achieved higher *Kappa*, higher accuracies in 15 and 30 e/h when estimating the global AHI, as well as in 5 and 15 e/h when estimating the central AHI, higher specificity in the three thresholds, and more balanced Se–Sp pairs in 5 and 15 e/h for both cases. Thus, our 2D-CNN architecture would be more effective than the ABD-based LSTM to simplify the processing of a recording aiming to establish a sleep apnea diagnosis and its severity.

As previously mentioned, several studies conducted individual or joint analyzes of THO and ABD signals to help automatically diagnose sleep apnea, but from an event-detection-based approach. In contrast, recent studies estimated the AHI by analyzing different physiological recordings from the SHHS, such as AF, SpO_2_, or ECG [49,55,56,57,58,59]. Despite the promising results achieved by these studies (84.30–95.70% Acc for 5 e/h, 81.10–91.00% Acc for 15 e/h, and 89.20–96.70% Acc for 30 e/h), the use of these cardiorespiratory signals limits the distinction between different types of apneic events. Both obstructive and central apnea result in the cessation of AF. However, the characteristic lack of respiratory effort during central events cannot be detected by this signal alone. Similarly, only oximetry data cannot differentiate between obstructive and central events. Both types of sleep apnea can lead to blood oxygen desaturation, necessitating additional information, such as respiratory effort signals, to make this distinction. Likewise, significant changes in heart activity can be caused by other coexisting conditions [52], so ECG alone cannot differentiate between obstructive and central events. Therefore, as recommended by the AASM [4], the use of THO and/or ABD signals becomes indispensable for identifying central apnea. It is important to highlight that distinguishing between these two types of sleep apnea is crucial for providing personalized and effective treatment.

Consequently, this CNN model could have significant clinical applications in the field of sleep medicine and healthcare. The proposed approach has demonstrated the potential for the automated diagnosis of sleep apnea using THO and ABD movement signals. This could significantly streamline the diagnosis process and reduce the need for extensive polysomnography, which often involves cumbersome equipment and overnight stays in hospital sleep labs. Moreover, an automated estimation of sleep apnea severity could lead to increased efficiency in diagnosing patients. Thereby, physicians could use our CNN model to screen patients more quickly, allowing for faster intervention and treatment than the standard diagnostic method. To this we should add that our proposal not only estimates the global AHI but also evaluates the contribution of the central respiratory events. This distinction is important in tailoring treatment approaches, as central and obstructive sleep apnea may require different interventions. By accurately estimating the severity of sleep apnea and differentiating central events, medical specialists could offer more personalized and effective treatments. This would be especially important for those subjects severely affected by the disease, since they are the ones at greatest risk of developing comorbidities and serious adverse consequences for their health [6,7,8]. Accordingly, sleep apnea affected subjects could receive interventions that are tailored to their specific condition, potentially leading to better outcomes and an improvement in their life quality.

However, this study has limitations that deserve mention. Although the SHHS public database is widely used for research related to sleep and cardiovascular health, it has some inconsistencies that could have negatively influenced the diagnostic performance of our proposed models. In this regard, there is a lack of agreement between the annotations of respiratory events (central apneas, obstructive apneas and hypopneas, desaturations, arousals, etc.) and the clinical indices derived from the sleep studies. As Karhu et al. (2021) stated [37], this could be due to data corruption over time, as well as changes in the clinical recommendations and the scoring criteria on which the SHHS sleep studies were based. This fact could have particularly affected the hypopneic event estimations and, therefore, the prediction of the sleep apnea severity in our study. In order to deal with this limitation, we have taken the direct event annotations provided for each subject by the clinicians as reference. Another main limitation of the study is the existing imbalance between the SHHS severity groups. While the predominant class (moderate apnea) contained 36.37% of the total, the minority class (no apnea) only contained 4.09% of the subjects. It should be considered that neural networks tend to favor the majority classes, reducing the performance in the classification of the minority classes. Thus, we applied data augmentation techniques to balance the severity groups in our study and overcome this particular drawback. However, this sampling method might not have been sufficient to correct the large imbalance in the dataset used to estimate the central AHI (8091 subjects with a central AHI less than 5) and test accuracy in under- or over-sampled conditions.

One promising avenue for future research would be to apply our model to other independent databases. This would allow us to explore the inherent variability in clinical practice (different sociodemographic and clinical factors, devices, and processes of signal acquisition, etc.) and broaden the scope of our findings. By testing our model on diverse datasets from different healthcare institutions and/or regions, we could assess its robustness and generalizability in real clinical settings. This not only would validate the effectiveness of our proposal but also would ensure that it remains reliable across several patient populations and clinical scenarios. Moreover, addressing class imbalance remains a priority in our research. Looking ahead to future lines of research, we point out the ongoing importance of mitigating the class imbalance, particularly for the central AHI dataset. Accordingly, we plan to explore several data balancing techniques, such as synthetic minority over-sampling technique (SMOTE) [60]. By rectifying the notable class imbalance in the dataset used for the central AHI estimation, we could ensure that our model does not exhibit biases towards the majority class. This would not only enhance the fairness of our CNN but also contribute to its generalizability, making it more reliable for a broader range of patients. Furthermore, there is room for innovation in the field of deep learning methods for estimating the AHI. While the proposed model leverages CNNs, exploring alternative architectures, like recurrent neural networks (RNNs), transformers, or hybrid models, could yield novel insights and potentially improve AHI estimation accuracy. Each of these architectures has its unique strengths, and their application could lead to a more comprehensive understanding of sleep apnea patterns, especially in complex cases. It should also be noted that deep learning methods have proven to be a powerful technique to help in the automated diagnosis of diseases, but also that they present a high difficulty when interpreting their results. Deep learning models, such as CNNs, are often seen as black boxes. As the model complexity increases, understanding how and why it makes certain decisions can be challenging. This can be a drawback in applications where explainability and transparency in the decision-making process are required. Consequently, we propose as a future line of investigation to apply our model together with explainable artificial intelligence (XAI) techniques to understand its predictions and improve its reliability.

## 5. Conclusions

This study focused on the development of a CNN architecture fed with respiratory effort data to estimate sleep apnea severity in a large adult population. The proposed methodology showed a high diagnostic performance estimating the global and central AHI of the subjects under study. These results suggest that THO and ABD are useful for estimating apnea severity by themselves, without the need for additional parameters to be included. Our findings also demonstrated that the physiological particularities of obstructive and central apnea as reflected in the respiratory effort signals are more clearly identified when the nature of the events is central. This fact led us to go a step further and establish intermediate AHI thresholds to comprehensively assess central sleep apnea. This evaluation revealed the great potential of our proposed model to effectively detect central events. Moreover, our CNN approaches outperformed a LSTM deep learning method that had previously been applied to respiratory effort recordings and aimed to estimate the AHI. This suggests that the information extracted from THO and ABD using a CNN architecture could be a promising and powerful tool to help diagnose sleep apnea, particularly in subjects suffering from central apnea.

## Figures and Tables

**Table 1:** Summary of the sociodemographic data from the participants.

Data	All	Training	Validation	Test
#Subjects	8257	2606 (31.56%)	651 (7.88%)	5000 (60.56%)
Age (years)	65 [16]	64 [21]	62 [12]	65 [16]
#Male	3887 (47.08%)	1275 (48.93%)	322 (49.46%)	2290 (45.80%)
BMI (kg/m^2^)	27.54 [6.10]	27.15 [6.18]	28.02 [6.32]	27.68 [6.02]
CAI (e/h)	0 [0.26]	0 [0.33]	0.12 [0.26]	0 [0.26]
AHI (e/h)	21.68 [21.52]	25.62 [23.08]	26.08 [26.48]	19.38 [19.51]
#No apnea	338 (4.09%)	77 (2.95%)	11 (1.69%)	250 (5.00%)
#Mild	2244 (27.18%)	529 (20.30%)	143 (21.97%)	1572 (31.44%)
#Moderate	3003 (36.37%)	958 (36.76%)	214 (32.87%)	1831 (36.62%)
#Severe	2672 (32.36%)	1042 (39.99%)	283 (43.47%)	1347 (26.94%)

BMI: Body mass index; CAI: central apnea index; AHI: apnea hypopnea index.

**Table 2:** Search space for the CNN hyperparameters.

Hyperparameter	Search Space	Optimal Value
$N_{c}$	6, 8	8
$S_{k}$	32, 64, 128 ($N_{c}=6$), 16, 32 ($N_{c}=8$)	16
$N_{k}$	16, 32, 64, 128	128
$p_{drop}$	0.1, 0.2, 0.3, 0.4	0.3

**Table 3:** Diagnostic performance of the CNN models proposed for the prediction of the global and central AHI in the test set (CNN-AHI_Global_ and CNN-AHI_Central_, respectively).

	AHI events/h	Se (%)	Sp (%)	Acc (%)	PPV (%)	NPV (%)	LR+	LR-	*kappa*	Acc_4_ (%)
**CNN-AHI_Global_**	5	99.09	16.80	94.98	95.77	49.41	1.19	0.05	0.3960	58.88
	15	88.58	64.54	79.82	81.33	76.41	2.50	0.18		
	30	66.74	87.08	81.60	65.57	87.66	5.17	0.38		
**CNN-AHI_Central_**	1	59.87	97.70	94.10	73.26	95.86	26.05	0.41	0.5903 **	98.50 **
	5	63.54	99.41	98.72	67.78	99.29	107.45	0.37		
	10	68.57	99.86	99.64	77.42	99.78	486.37	0.31		
	15	45.45	99.98	99.74	90.91	99.76	2262.72	0.55		
	30 *	-	-	-	-	-	-	-		

* No subjects with ≥30 central events/h. ** Computed for 3 classes from its confusion matrix.

**Table 4:** Comparison with other state-of-the-art studies based on the analysis of THO and/or ABD by means of feature engineering or deep learning techniques.

Study	#Subjects	Signal	Approach	AHI	Se (%)	Sp (%)	Acc (%)	*Kappa*
**Ng et al. (2008)** [21]	26	THO, ABD	Event detection by temporal features	-	88.29	90.19	89.20 **	-
**Lin et al. (2017)** [18]	34	THO, ABD	Event detection by temporal and frequency features	-	-	-	84.42	-
**Varady et al. (2003)** [20]	6	THO, ABD	Event detection by phase features	-	-	-	90.63	-
**Al-Angari et al. (2012)** [22]	100 (SHHS)	THO, ABD	Event detection by phase features	-	85.7	92.20	89.00	-
**Guijarro-Berdiñas et al. (2012)** [23]	6	AF, THO, ABD	Obstructive event detection by wavelet features	-	89.63	97.11	94.62	-
			Central event detection by wavelet features	-	88.98	98.71	95.47	-
			Mixed event detection by wavelet features	-	92.20	89.58	90.45	-
**Sezgin et al. (2009)** [24]	21	THO, ABD	Obstructive, central, and mixed event distinction by wavelet features	-	-	-	86.84	-
**Emin et al. (2010)** [25]	21	ABD	Obstructive, central, and mixed event distinction by wavelet features	-	-	-	75.85	-
**Fontenla-Romero et al. (2005)** [26]	6	AF, THO	Obstructive, central, and mixed event distinction by wavelet features	-	-	-	83.78	-
**Koley et al. (2013)** [28]	20	AF, THO	Obstructive, central, and mixed event distinction by wavelet features	-	-	-	85.00	-
**Avci et al. (2015)** [29]	8	ABD	Event detection by wavelet features	-	-	-	92.75	0.9250
		THO	Event detection by wavelet features	-	-	-	95.87	0.9360
**Haidar et al. (2018)** [34]	2056	AF, THO, ABD	Event detection using CNN	-	-	-	83.50	-
**Wang et al. (2023)** [33]	1507	AF, THO, ABD	Event detection using CNN	-	77.14	86.19	81.67	-
			Event detection using LSTM	-	77.68	84.43	81.56	-
			Event detection using 1D-CNN-LSTM	-	81.73	85.30	83.53	-
			Event detection using 2D-CNN-LSTM	-	81.21	86.59	83.90	-
**Van Steenkiste et al. (2019)** [32]	2100 (SHHS)	ABD	AHI estimation using a LSTM method to detect apneic events	5	99.34	5.88	97.75	0.3293
				15	91.04	37.61	78.76	
				30	67.19	81.51	76.01	
**Our proposal**	**8257**	**THO, ABD**	**Global AHI estimation using a CNN method**	**5**	**99.09**	**16.80**	**94.98**	**0.3960**
				**15**	**88.58**	**64.54**	**79.82**	
				**30**	**66.74**	**87.08**	**81.60**	
			**Central AHI estimation using a CNN method**	**5**	**63.54**	**99.41**	**98.72**	**0.5903**
				**15**	**45.45**	**99.98**	**99.74**	
				30 *	**-**	**-**	**-**	

* No subjects with ≥30 central events/h. ** Computed from reported data. AF: Airflow signal; THO: thoracic signal; ABD: abdominal signal; LSTM: long short-term memory neural network; CNN: convolutional neural networks.

## Data Availability

The data presented in this research can be obtained by contacting the corresponding author. As a result of safeguarding the privacy of the participants, the data is not accessible to the public.

## Supplementary Materials

The following supporting information can be downloaded at: https://www.mdpi.com/article/10.3390/diagnostics13203187/s1. Table S1. Confusion matrix obtained by the CNN model for the severity prediction from the global AHI (CNN-AHI_Global_) in the SHHS1 set. Table S2. Confusion matrix obtained by the CNN model for the severity prediction from the global AHI (CNN-AHI_Global_) in the SHHS2 set. Table S3. Confusion matrix obtained by the CNN model for the severity prediction from the central AHI (CNN-AHI_Central_) in the SHHS1 set. Table S4. Confusion matrix obtained by the CNN model for the severity prediction from the central AHI (CNN-AHI_Central_) in the SHHS2 set. Table S5. Diagnostic performance of the CNN models proposed for the prediction of the global and central AHI in the SHHS1 set (CNN-AHI_Global_ and CNN-AHI_Central_, respectively). Table S6. Diagnostic performance of the CNN models proposed for the prediction of the global and central AHI in the SHHS2 set (CNN-AHI_Global_ and CNN-AHI_Central_, respectively).
