# Supplementary material for: Prediction of the Sleep Apnea Severity Using 2D-Convolutional Neural Networks and Respiratory Effort Signals
Source: Diagnostics (Basel). 2023 Oct 12;13(20):3187. doi: 10.3390/diagnostics13203187 (PMC10605440; doi:10.3390/diagnostics13203187)
Supplement: Supplementary file 1 [file diagnostics-13-03187-s001.zip › diagnostics-2509477-supplementary.pdf]

SHHS1 – Global AHI		CNN-AHI$_{Global}$			
		No apnea	Mild apnea	Moderate apnea	Severe apnea
PSG	No apnea	8	46	3	0
	Mild apnea	16	340	166	4
	Moderate apnea	3	243	604	101
	Severe apnea	2	70	357	537

Table S1. Confusion matrix obtained by the CNN model for the severity prediction from the global AHI (CNN-AHI$_{Global}$) in the SHHS1 set.

SHHS2 – Global AHI		CNN-AHI$_{Global}$			
		No apnea	Mild apnea	Moderate apnea	Severe apnea
PSG	No apnea	34	150	8	1
	Mild apnea	21	561	435	29
	Moderate apnea	1	44	498	337
	Severe apnea	0	0	19	362

Table S2. Confusion matrix obtained by the CNN model for the severity prediction from the global AHI (CNN-AHI$_{Global}$) in the SHHS2 set.

SHHS1 – Central AHI		CNN-AHI$_{Central}$		
		No apnea	Mild apnea	Moderate apnea
PSG	No apnea	2448	16	0
	Mild apnea	12	16	0
	Moderate apnea	1	6	1

Table S3. Confusion matrix obtained by the CNN model for the severity prediction from the central AHI (CNN-AHI$_{Central}$) in the SHHS1 set. No subjects with ≥30 central events/h.

SHHS2 – Central AHI		CNN-AHI$_{Central}$		
		No apnea	Mild apnea	Moderate apnea
PSG	No apnea	2427	13	0
	Mild apnea	21	24	1
	Moderate apnea	1	4	9

Table S4. Confusion matrix obtained by the CNN model for the severity prediction from the central AHI (CNN-AHI$_{Central}$) in the SHHS2 set. No subjects with ≥30 central events/h.

SHHS1	AHI events/h	Se (%)	Sp (%)	Acc (%)	PPV (%)	NPV (%)	LR+	LR-	*kappa*	Acc$_4$ (%)
CNN-AHI$_{Global}$	5	99.14	14.04	97.20	98.02	27.59	1.15	0.06	0.3962	59.56
	15	83.41	70.33	80.36	90.24	56.32	2.81	0.24		
	30	55.59	93.16	78.64	83.64	76.91	8.12	0.48		
CNN-AHI$_{Central}$	1	53.25	98.10	93.96	74.10	95.37	28.10	0.48	0.5271**	98.60**
	5	63.89	99.35	98.84	58.97	99.47	98.39	0.36		
	10	66.67	99.92	99.76	80.00	99.84	829.33	0.33		
	15	12.50	100.00	99.72	100.00	99.72	Inf.	0.88		
	30*	-	-	-	-	-	-	-		

Table S5. Diagnostic performance of the CNN models proposed for the prediction of the global and central AHI in the SHHS1 set (CNN-AHI$_{Global}$ and CNN-AHI$_{Central}$, respectively). * No subjects with ≥30 central events/h. ** Computed for 3 classes from its confusion matrix.

SHHS2	AHI events/h	Se (%)	Sp (%)	Acc (%)	PPV (%)	NPV (%)	LR+	LR-	*kappa*	Acc$_4$ (%)
CNN-AHI$_{Global}$	5	99.05	17.62	92.76	93.49	60.71	1.20	0.05	0.3962	58.20
	15	96.43	61.82	79.28	72.00	94.45	2.53	0.06		
	30	95.01	82.68	84.56	49.66	98.93	5.49	0.06		
CNN-AHI$_{Central}$	1	66.12	97.29	94.24	72.65	96.35	24.44	0.35	0.6333**	98.40**
	5	63.33	99.47	98.60	74.51	99.10	118.87	0.37		
	10	69.57	99.80	99.52	76.19	99.72	344.63	0.31		
	15	64.29	99.96	99.76	90.00	99.80	1598.14	0.36		
	30*	-	-	-	-	-	-	-		

Table S6. Diagnostic performance of the CNN models proposed for the prediction of the global and central AHI in the SHHS2 set (CNN-AHI$_{Global}$ and CNN-AHI$_{Central}$, respectively). * No subjects with ≥30 central events/h. ** Computed for 3 classes from its confusion matrix.
